# Proteomic Changes Induced by the Immunosuppressant Everolimus in Human Podocytes

**DOI:** 10.3390/ijms25137336

**Published:** 2024-07-04

**Authors:** Maurizio Bruschi, Simona Granata, Giovanni Candiano, Andrea Petretto, Martina Bartolucci, Xhuliana Kajana, Sonia Spinelli, Alberto Verlato, Michele Provenzano, Gianluigi Zaza

**Affiliations:** 1 Laboratory of Molecular Nephrology, IRCCS Istituto Giannina Gaslini, 16147 Genoa, Italy; mauriziobruschi@gaslini.org (M.B.); giovannicandiano@gaslini.org (G.C.); xhulianakajana@gaslini.org (X.K.); soniaspinelli@gaslini.org (S.S.); 2 Department of Experimental Medicine (DIMES), University of Genoa, 16132 Genoa, Italy; 3 Department of Medical and Surgical Sciences, University of Foggia, 71122 Foggia, Italy; simona.granata@unifg.it; 4 Proteomics and Clinical Metabolomics Unit at the Core Facilities, IRCCS Istituto Giannina Gaslini, 16147 Genoa, Italy; andreapetretto@gaslini.org (A.P.); martinabartolucci@gaslini.org (M.B.); 5 Renal Unit, Department of Medicine, University Hospital of Verona, 37124 Verona, Italy; alberto.verlato@univr.it; 6 Nephrology, Dialysis and Transplantation Unit, Department of Pharmacy, Health and Nutritional Sciences, University of Calabria, 87036 Rende, Italy; michele.provenzano@unical.it

**Keywords:** mTOR-inhibitors, everolimus, podocytes, kidney transplantation, proteomics

## Abstract

mTOR inhibitors (mTOR-Is) may induce proteinuria in kidney transplant recipients through podocyte damage. However, the mechanism has only been partially defined. Total cell lysates and supernatants of immortalized human podocytes treated with different doses of everolimus (EVE) (10, 100, 200, and 500 nM) for 24 h were subjected to mass spectrometry-based proteomics. Support vector machine and partial least squares discriminant analysis were used for data analysis. The results were validated in urine samples from 28 kidney transplant recipients receiving EVE as part of their immunosuppressive therapy. We identified more than 7000 differentially expressed proteins involved in several pathways, including kinases, cell cycle regulation, epithelial–mesenchymal transition, and protein synthesis, according to gene ontology. Among these, after statistical analysis, 65 showed an expression level significantly and directly correlated with EVE dosage. Polo-Like Kinase 1 (PLK1) content was increased, whereas osteopontin (SPP1) content was reduced in podocytes and supernatants in a dose-dependent manner and significantly correlated with EVE dose (*p* < 0.0001, FDR < 5%). Similar results were obtained in the urine of kidney transplant patients. This study analyzed the impact of different doses of mTOR-Is on podocytes, helping to understand not only the biological basis of their therapeutic effects but also the possible mechanisms underlying proteinuria.

## 1. Introduction

The mTOR inhibitors (mTOR-Is) everolimus and sirolimus are drugs used as part of immunosuppressive therapy in kidney transplantation. They exert their mechanisms of action by regulating mTORC1 and 2, two multi-subunit complexes involved in several cellular pathways. mTORC1 promotes anabolic cellular processes by stimulating the synthesis of proteins, lipids, and nucleotides and, at the same time, inhibits catabolic processes, such as lysosome biogenesis and autophagy. mTORC2 controls cell survival, cytoskeleton organization, lipogenesis, and gluconeogenesis [1,2].

However, these drugs are associated with a significantly higher incidence of de novo or exacerbated proteinuria [3,4]. High proteinuria appears to be more common when mTOR-I is initiated at a later time point after kidney transplantation (1 year) and in the presence of proteinuria >0.8 g/day at the time of the switch from calcineurin inhibitors (CNI) [5,6]. Letavernier et al. observed the development of proteinuria in more than 50% of patients after conversion, some of them having nephrotic-range proteinuria [7]. Similarly, Morelon and Kreis reported the development of proteinuria in 32/50 patients, together with nephrotic syndrome in 18 cases after switching from CNI to sirolimus [8].

The mechanism by which mTOR-Is cause proteinuria, particularly after conversion from a CNI-based regimen, remains unknown. Several mechanisms have been proposed, such as an increase in intra-glomerular pressure with a concomitant reduction of kidney reserve and glomerular hyperfiltration [9], a reduction in vascular endothelial growth factor (VEGF) synthesis resulting in podocyte injury [10], and altered protein endocytosis in tubular epithelial cells [11].

In kidney transplant biopsies performed prior to and 2 years after conversion from CNI to sirolimus, Stallone et al. found that slit diaphragm (SD)-associated protein expression (nephrin, podocin, and CD2ap) was downregulated 24 months after conversion and that sirolimus blood trough levels were inversely correlated with SD protein expression [12], demonstrating a dose-dependent effect of the drug on podocyte structure.

However, the mechanisms underlying podocyte injury leading to mTOR inhibitor-induced proteinuria are only partially defined.

The aim of this study was to evaluate, through an untargeted proteomic approach (based on innovative bioinformatic algorithms), the protein expression profiles of podocytes and their supernatants after in vitro treatment with different doses of everolimus (10, 100, 200 and 500 nM). The results of proteomic analysis were then validated in an independent cohort of kidney transplant recipients undergoing everolimus-based maintenance immunosuppressive therapy.

High-throughput strategies may help add new insights into the molecular mechanisms underlying this effect and identify factors associated with these conditions that could be useful as disease progression biomarkers and novel therapeutic targets.

## 2. Results

### 2.1. Proteomic Profile

In podocytes, 7786 proteins were identified, and among these, 7363 (94.6%) were common in all conditions (Tables S1 and S2). Only 13 (0.17%), 5 (0.06%), 14 (0.18%), 12 (0.15%), and 10 (0.13%) proteins were exclusive to CTR or cells treated with 10, 100, 200, and 500 nM everolimus, respectively (Figure S1A and Table S2). Interestingly, 6.2%, 29.8%, 22.1%, 22.3%, and 19.6% were annotated as extracellular, membrane, nucleus, cytoplasm, and organelle proteins, respectively.

In supernatants, 4494 proteins were identified, among which 2508 (55.8%) were common in all conditions (Tables S1 and S3). Only 204 (4.5%), 205 (4.6%), 87 (1.9%), 107 (2.4%), and 156 (3.5%) proteins were exclusive to the supernatants of cells untreated (CTR) or treated with 10, 100, 200, and 500 nM everolimus, respectively (Figure S1B and Table S3). Among the total proteins identified in the supernatants, 27%, 24.1%, 16.7%, 18.5%, and 13.7% were annotated as extracellular, membrane, nucleus, cytoplasm, and organelle proteins, respectively.

Finally, we recognized 274 (3.5%) kinases in podocytes and 174 (3.9%) in supernatants (Table S4).

An ANOVA test for paired samples was used to identify proteins, in cells and their supernatants, able to discriminate CTR and the four treatments. A total of 939 proteins were identified, 630 (67.1%) and 360 (33.3%) of which were statistically significant in cells (Table S5) and their supernatants (Table S6), respectively. Partial least squares discriminant analysis (PLS-DA) and support vector machine (SVM) learning analyses were performed to reduce the number of selected proteins that maximized discrimination among all conditions and to prioritize their importance. These analyses identified a ranked core panel of 65 proteins (Table S7). Their priority was determined using the rank list and the variable importance in projection (VIP) score obtained using SVM and PLS-DA, respectively. The discrimination of the 10 conditions corresponded to a VIP score limit of 1.3899. Both analyses identified the same protein priority order. The expression profile of this core panel of proteins after Z-score normalization is visualized in the heatmap shown in Figure S2. This core panel of 65 proteins was also correlated with EVE dose (Table S7).

**Figure 1:**
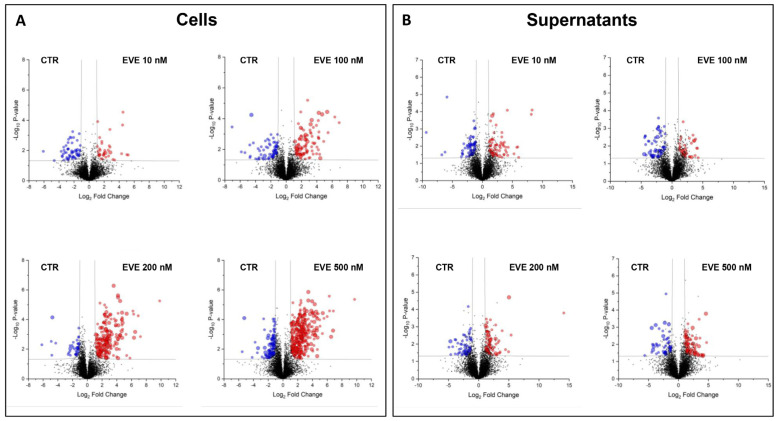
Volcano plots of the comparison between untreated and everolimus (EVE)-treated (**A**) podocytes and (**B**) their supernatants. Graphical representation of T-test analysis applied to the entire cell proteomic dataset. In the volcano graph, the x and y axes show the change in protein profiles between untreated (CTR) and treated with 10 nM EVE, CTR and treated with 100 nM EVE, CTR and 200 nM EVE, and CTR and 500 nM EVE and their −Log10 *p*-values, respectively. The black curves indicate the statistical significance threshold. Black, red, and blue circles correspond to non-statistically significant proteins or statistically significant proteins that were upregulated or downregulated in treated samples, respectively.

Next, to identify the proteins able to discriminate cells and their supernatants between CTR and each treatment, we used the *t*-test. Of the 630 proteins identified by ANOVA in cells, 92, 163, 244, and 440 were statistically significant in the comparison of CTR vs. 10 nM, CTR vs. 100 nM, CTR vs. 200 nM, and CTR vs. 500 nM conditions, respectively (Table S5 and Figure S3A). Of the 360 proteins identified by ANOVA in supernatants, 123, 93, 118, and 119 were statistically significant in the comparison of CTR vs. 10 nM, CTR vs. 100 nM, CTR vs. 200 nM, and CTR vs. 500 nM conditions, respectively (Table S6 and Figure S3B). Figure 1 shows a graphical representation of all *t*-test comparisons of cells and their supernatants. Of the 939 statistically significant proteins identified, only 224 and 163 were correlated with the dose of EVE in cells and their supernatants, respectively (Figure S4).

### 2.2. Gene Ontology Enrichment Analysis

**Figure 2:**
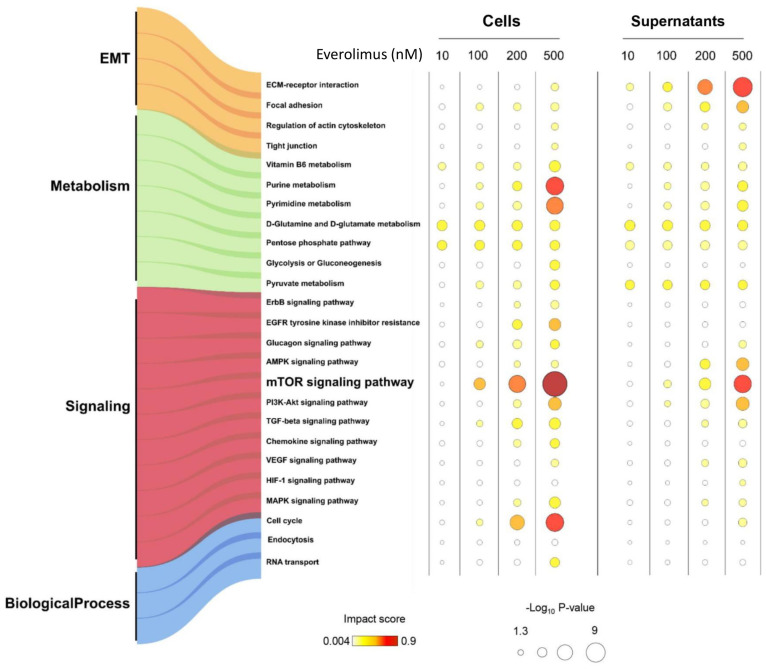
Gene ontology (GO) enrichment analysis bubble diagram. Bubble diagram of 25 GO terms enriched in cells and supernatants. In the diagram, each row represents a GO term, and each column corresponds to a condition. The normalized impact scores are represented by a pseudocolor scale varying between white, yellow, and red, corresponding to the minimum, median, and maximum impact score values, while the size was proportional to the −Log10 *p*-value.

To assess the biological roles of the proteins identified, we performed an integrated gene ontology (GO) annotation enrichment analysis using all the proteins identified. This analysis revealed that the differentially expressed proteins were responsible for the perturbation of 25 GO annotation terms that can be grouped into four main biological processes, including epithelial-to-mesenchymal transition, metabolism, kinase signaling, and other processes (see details in Table S8 and Figure 2).

**Figure 3:**
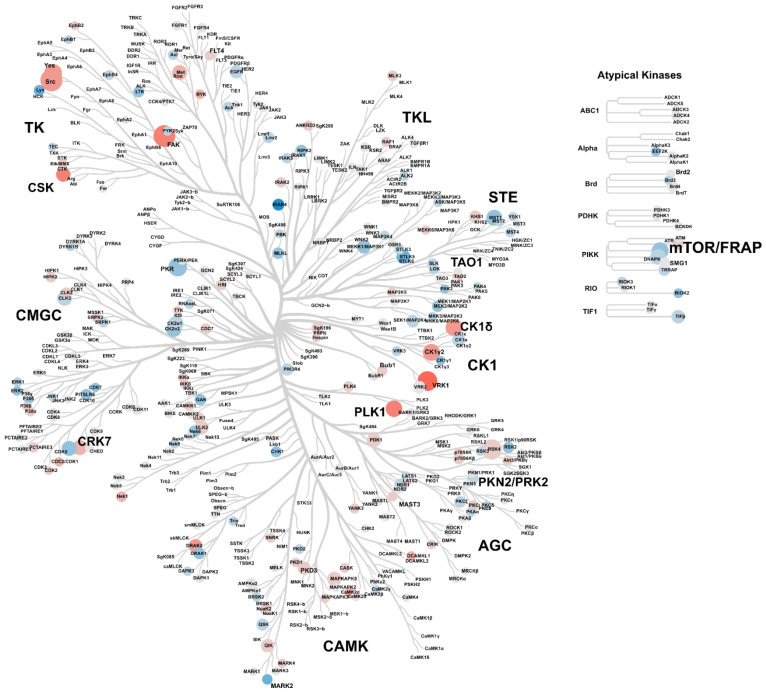
Kinase tree diagram generated using the Coral app for comparison between podocytes untreated and treated with 500 nM EVE. Each circle represents an identified kinase. Log2 kinase fold change is depicted by a pseudocolor scale, with red indicating upregulation, white equal expression, and blue downregulation in treated samples. The circle size was proportional to the corresponding −Log10 *p*-value in the *t*-test.

In addition, considering the biological role of EVE, we performed a kinase enrichment analysis (KEA) using all of the significant proteins identified to highlight the dysregulation of phosphorylation signaling induced by the drug. Of the 275 kinases identified, 6 were statistically significant in at least one comparison (Table S4 and Figures S5–S11). Interestingly, we found a significant upregulation of several kinases in cells after treatment with high-dose EVE (Figure 3) and, although this increment does not necessary imply an increase in the activity of the enzymes, it represents an important alteration in the kinase signaling landscape.

**Figure 4:**
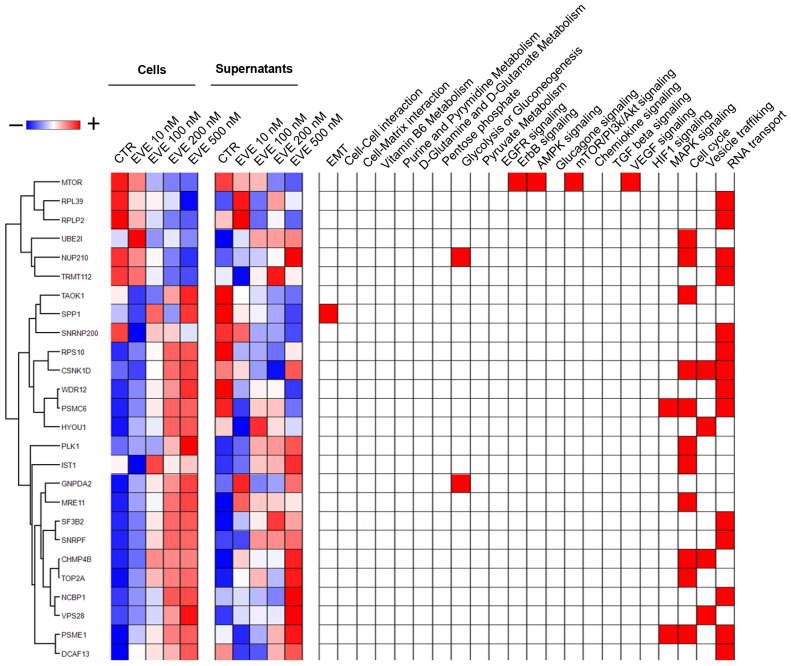
Heatmap of 26 proteins highlighted by integration of statistical and gene ontology (GO) enrichment analysis. In the heatmap, each row represents a protein, and each column corresponds to a condition. Normalized Z scores for protein abundance are represented by a pseudocolor scale, with red indicating positive expression and blue indicating negative expression relative to each protein value. The dendrogram positioned above and to the left of the heatmap shows the results of the unsupervised hierarchical clustering analysis, which placed similar protein profile values close to each other. The diagram positioned to the right of the heatmap indicates proteins associated with enriched GO (red).

Finally, the integration of all statistical and bioinformatic analyses, including ANOVA, *t*-test, PLS-DA, and SVM algorithms, highlighted 26 proteins that were significantly correlated with the EVE dose and associated with at least one of the biological processes identified by the GO enrichment analysis (Figure 4).

**Figure 5:**
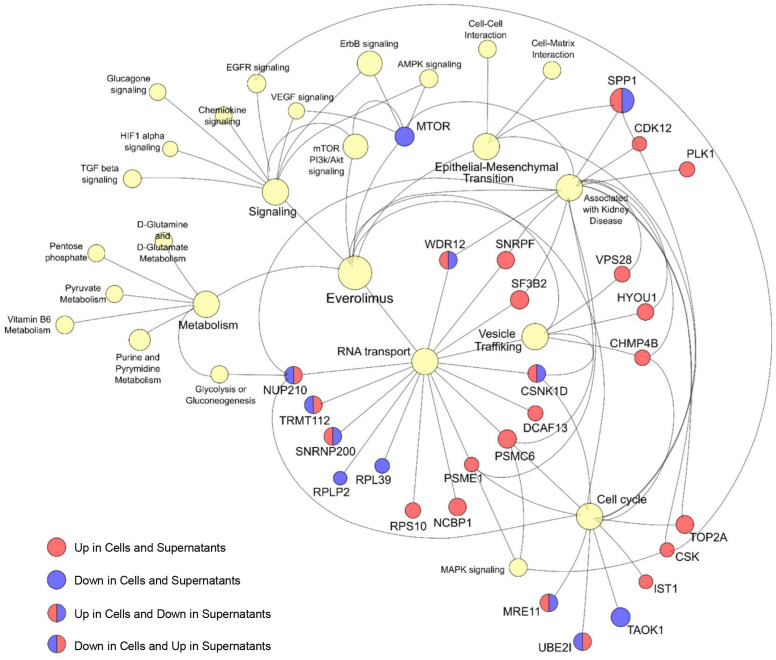
Protein interaction network. The diagram shows the association between the 28 highlighted proteins and gene ontology (GO) annotation terms as a network. Nodes (circle) and edges (line) represent proteins/GO terms and their interactions, respectively. The color intensity of each protein (node) indicates upregulation (red) or downregulation (blue) in cells or supernatants. The light yellow nodes represent GO terms.

Figure 5 shows the association between the 26 discriminating proteins and the GO annotation terms as a network diagram.

### 2.3. AKT-mTOR Phosphorylation Pathway Profiling

**Figure 6:**
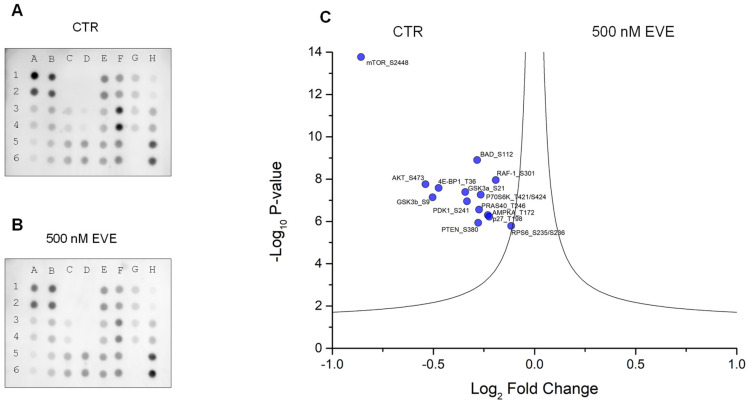
AKT-mTOR phosphorylation pathway profiling array. Podocytes untreated (CTR) (**A**) or treated with 500 nM everolimus (EVE) (**B**) were lysed, and the cell lysates were subjected to phosphorylation pathway profiling array. In the array, the spots A1, B1, A2, B2, H5, and H6 correspond to positive controls; C1, D1, C2, D2, G5, and G6 correspond to negative controls; A3, A4, F3, F4, E5, E6, F5, and F6 correspond to internal controls; E1, E2 to AKT S473; F1, F2 to AMPKa T172; G1, G2 to BAD S112; H1, H2 to 4E-BP1 T36; B3, B4 to GSK3a S21; C3, C4 to GSK3b S9; D3, D4 to mTOR S2448; E3, E4 to p27 T198; G3, G4 to P70S6K T421/S424; H3, H4 to PDK1 S241; A5, A6 to PRAS40 T246; B5, B6 to PTEN S380; C5, C6to RAF-1 S301; D5, D6 to RPS6 S235/S236. (**C**) Graphical representation of Mann-Whitney analysis applied to the phosphorylation pathway profiling array. In the volcano graph, the x and y axes, respectively, show the change in intensity profile between CTR and podocytes treated with 500 nM EVE and their -Log10 *p*-value. The black lines indicate the statistical significance threshold. Blue circles correspond to significantly downregulated phosphorylation in treated samples.

To assess the downregulation of the AKT-mTOR pathway by the EVE treatment, we performed phosphorylation pathway profiling using the Human Phosphorylation Pathway Profiling Array C55 kit. Figure 6A,B shows that AKT-mTOR phosphorylation signaling was significantly reduced by the EVE treatment. Interestingly, this array can detect the relative levels of phosphorylation of 55 proteins involved in several signaling pathways. Figure 6C shows that the phosphorylation signals of p-AKT, pAMPKa, p-BAD, p-4E-BP1, p-GCK3a, p-GSK3b, p-mTOR, p-p27, p-P70S6K, p-PDK1, p-PRAS40, p-PTEN, p-RAF-1, and p-RPS6 proteins were significantly reduced in podocytes treated with 500 nM EVE compared with CTR. The cells were treated with this drug dose based on the proteomic results, showing major protein deregulation. Moreover, 500 nM EVE was not associated with a high degree of cytotoxicity (Figure S14).

### 2.4. PLK1 Phosphorylation Activity

**Figure 7:**
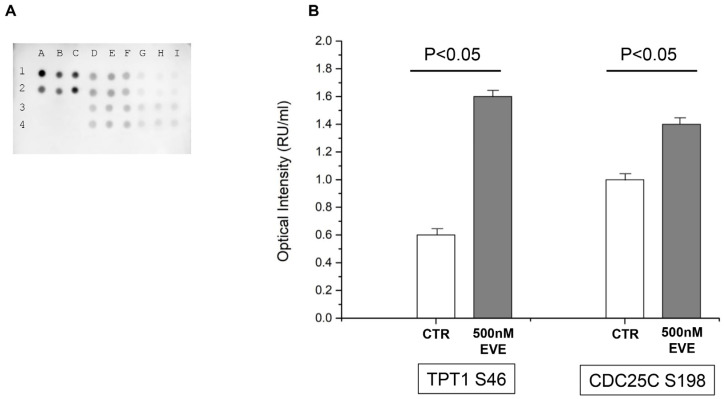
PLK1 Phosphorylation activity. (**A**) Dot-blot assay of PLK1 phosphorylation activity. In the dot-blot, the spots A1, B1, C1, A2, B2, and C2 correspond to positive controls; A3, B3, C3, A4, B4, and C4 to negative controls; D1, E1, F1, D2, E2, and F2 to TPT1 S46 of everolimus-treated cells (500 nM EVE); G1, H1, I1, G2, H2, and I2 to TPT1 S46 of untreated cells (CTR); D3, E3, F3, D4, E4, and F4 to CDC25C S198 of 500 nM EVE; G3, H3, I3, G4, H4, and I4 to CDC25C S198 of CTR. (**B**) Graphical representation of Mann–Whitney analysis applied to the phosphorylation of PLK1 substate (TPT1 and CDC25C). PLK1 activity on TPT1 and CDC25C proteins is significantly upregulated by EVE treatment.

To evaluate the activity of PLK1 in our cellular model, we used an immuno dot-blot assay with two of its substrates: translationally controlled tumor protein serine 46 (TPT1 S46) and M-phase inducer phosphatase 3 serine 198 (CDC25C S198). Figure 7 shows that both substrates were significantly increased in podocytes treated with 500 nM EVE compared with CTR (*p* < 0.05). In addition, the dose-related increase in PLK1 expression in extracellular vesicles and supernatants demonstrated the podocyte-active production of this protein (Figure S12).

### 2.5. Urinary Content of Polo-Like Kinase 1 (PLK1) and Osteopontin (SPP1)

**Figure 8:**
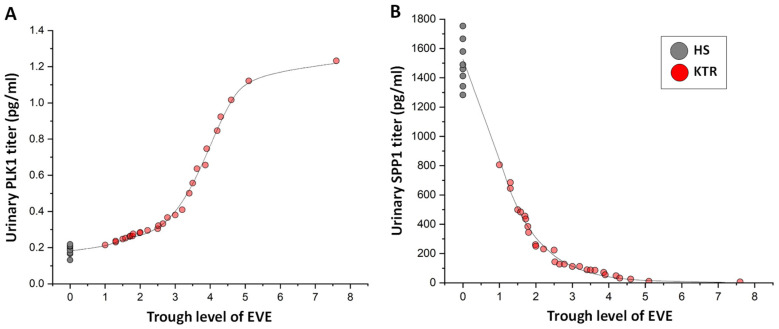
Correlation between urinary (**A**) PLK1 and (**B**) SPP1 levels and EVE trough levels in 28 kidney transplant recipients. Eight healthy subjects were included in the analysis. The black line indicates the best fitting equation model between the two features corresponding to (**A**) a sigmoid curve (R = 0.99) and (**B**) exponential decay (R = 0.99).

To validate the mass spectrometry results, we assessed the urine PLK1 and SPP1 contents of 28 kidney transplant recipients in the everolimus-based immunosuppressive regimen and 8 healthy volunteers matched for gender and age. The levels of both proteins were correlated with everolimus trough levels. In particular, the nonlinear regression fitting equation detector of OriginLab software (version 2022b) identified a sigmoid curve concordance between everolimus dose and urinary PLK1 (R = 0.99) and an exponential decay concordance between everolimus dose and urinary SPP1 content (R = 0.99) (Figure 8).

## 3. Discussion

The wide clinical use of mTOR-Is in kidney transplantation is currently limited by the high risk of adverse effects including edema, hematological adverse effects, pulmonary toxicity, and proteinuria. The latter, occurring in up to 45% of patients receiving mTOR-Is and generally dose-related, is associated with diminished patient and kidney graft survival [13].

Several mechanisms have been proposed to explain the onset of this complication, but the complete pathological machinery has only been partially defined. The damage appears to be of glomerular origin, and podocytes, as key components of the glomerular filtration barrier, could play a central role.

Our study, using a high-throughput methodology (mass spectrometry-based proteomics) associated with an innovative bioinformatics approach, analyzed the proteomic profiles (more than 7000 proteins) of podocytes and their supernatants treated with different doses of everolimus (10, 100, 200, and 500 nM) and validated these results in urine samples from 28 kidney transplant recipients undergoing everolimus-based maintenance immunosuppressive therapy.

Interestingly, as previously demonstrated in other cellular models [14,15,16], major protein deregulation was observed following treatment with high-dose EVE (500 nM). We speculate that this result could be due to the short in vitro treatment period for podocytes, whereas kidney transplant recipients need long-term treatment with this medication at a lower dosage.

Many proteins could discriminate the different dosages of EVE, and 65 core proteins were significantly correlated with the drug dose. The integrated gene ontology (GO) enrichment analysis of all proteins identified via statistical analysis demonstrated that they were primarily involved in epithelial-to-mesenchymal transition, metabolism, and kinase signaling.

In addition, kinase analysis identified six proteins: Casein Kinase 1 Delta, Cyclin Dependent Kinase 12, C-Terminal Src Kinase, mTOR, Polo-like kinase 1, and TAO Kinase 1. The most upregulated was Polo-like kinase 1 (PLK1), an evolutionarily conserved Ser/Thr kinase that plays an important role in cell cycle regulation and is mainly expressed in the G2/S and M phases of the cell cycle [17].

As previously reported, PLK1 was upregulated in the kidney tissue of patients with chronic kidney disease and mice which underwent unilateral ureteral obstruction. It participated in myofibroblast activation and promoted kidney fibrosis by regulating the TGF-β1 signaling pathway. In contrast, reduced PLK1 expression by shRNA or pharmacological treatment reduced kidney fibrosis, preserved tubular structure, and attenuated inflammation by regulating autophagy [18], a biological pathway associated with myofibroblast activation and fibrosis through matrix protein synthesis and degradation, intracellular component recycling, and extracellular vesicle secretion [19]. In starved cells, PLK1 activated autophagy by inhibiting MTORC1, stimulating autophagosome formation [20], and preserving lysosomal function [18]. When PKL1 was downregulated, the reduced activities of the lysosomal proton pump V-ATPase subunit (ATP6V1A) and the enzyme cathepsin B caused lysosome damage and autophagy impairment [18].

Several studies have shown that proteinuria caused by podocyte damage could be associated with impaired autophagic clearance and lysosome depletion, suggesting a protective role of these pathways in podocytes [21,22]. Moreover, the loss of the lysosomal proteinase cathepsin D in podocytes led to the accumulation of abundant autophagosomes/auto-lysosome-like bodies, which triggered apoptotic cell death followed by proteinuria and glomerulosclerosis [23].

In our cellular model, high PLK1 content and activity could mediate the activation of the autophagy pathway with a protective role against podocyte damage caused by high-dose mTOR-I.

Similarly, in the urine of kidney transplant recipients receiving this drug as part of their maintenance immunosuppressive therapy, we found a direct correlation between urinary PLK1 content and blood EVE trough levels.

We also found that osteopontin (SPP1), a secreted glycoprotein with pleiotropic functions, was upregulated in EVE-treated podocytes in a dose-dependent manner. This protein mediates numerous biological processes involved in biomineralization, cellular homeostasis, several chronic inflammatory diseases, and tumor development and progression [24].

The upregulation of SPP1 expression was previously associated with macrophage accumulation within the kidney, proteinuria, loss of renal function, and severe histological damage, including tubulitis and tubulointerstitial fibrosis [25,26]. However, SPP1 overexpression may also play a protective role against renal injury. The upregulation of its expression is associated with the regeneration and repair of tubular cells in the recovery process following tubular damage [27] and can alter the actin cytoskeleton to enhance the ability of these cells to resist stress such as mechanical strain [28].

In our podocyte cellular model, SPP1 protein content was reduced in the supernatants in a dose-dependent manner. We can speculate that this effect could be explained by the activation of intracellular signaling, leading to a reduction in SPP1 secretion in the attempt to accumulate this protein in the cytoplasm of podocytes and potentially hinder the damage induced by high-dose EVE.

Moreover, the dose-related variation in the protein level of PLK1 and SPP1 with a similar trend observed in both extracellular vesicles and supernatants suggests the active secretion of these proteins.

Also in the urine of kidney transplant patients, the high trough level of everolimus was associated with a low SPP1 content, probably confirming the in vitro result. However, the in vivo results for both PLK1 and SPP1 cannot completely exclude an extra-podocyte origin of these proteins. Additional studies are necessary to assess this point.

## 4. Materials and Methods

### 4.1. Reagents

Dulbecco’s modified Eagle’s medium (DMEM) high glucose, FBS, L-glutamine, penicillin, and streptomycin were purchased from Gibco (Waltham, MA, USA). Everolimus (purity: 99.78%) was purchased from Selleck Chemicals, (Houston, TX, USA) dissolved in DMSO, aliquoted, and stored at −20 °C until use. LYSE buffer was obtained from Preomics (Martinsried, Germany). The antibodies used in this study were PLK1 purchased from Biorbyt (Cambridge, UK) and secondary goat anti-mouse IgG HRP-conjugated antibody from Novus Biologicals (Centennial, CO, USA). All chemicals used for proteomic analysis were purchased from Thermo Fisher Scientific unless otherwise stated. All solutions were prepared with deionized water of resistivity no less than 18.2 MΩ cm^−1^.

### 4.2. Cells

An immortalized human podocyte cell line obtained via infection of primary cultures with a hybrid Adeno5/SV40 virus [29] was used in the experiments. Western blot analysis revealed that our podocyte cell line was positive for nephrin (NPHS1), podocin (NPHS2), and synaptopodin (SYNPO), showing a typical phenotype of human podocytes (Figure S13).

The cells were grown in Dulbecco’s modified Eagle’s medium (DMEM) high glucose supplemented with 10% FBS, 2 mM L-glutamine, penicillin (100 U/mL), and streptomycin (100 μg/mL).

Cells were seeded at a density of 2.5 × 10^5^/T25 flask and then exposed to 10, 100, 200, or 500 nM everolimus for 24 h. At the end of treatment, the cells and supernatants were collected and stored at −80 °C until use.

The MTT assay showed that everolimus induced cytotoxicity in a dose-dependent manner (Figure S14). Moreover, it reduced the expression of proteins of the slit diaphragm (Figure S15). All experiments were performed in triplicate.

### 4.3. Mass Spectrometry Profile

Cells were lysed, reduced, and alkylated in 50 µL LYSE buffer (Preomics, Martinsried, Germany) at 95 °C for 10 min at 1000 rpm. Lysates were digested with 0.7 µg Trypsin and 0.3 μg LysC overnight at 37 °C and then processed by iST protocol [30]. The cell supernatants were first cooled on ice, and sodium deoxycholate was added to a final concentration of 0.01% *w*/*v*. After mixing, TCA was added to a final 7.5% concentration, and the solution was precipitated on ice for 2 h. The mixed protein-detergent precipitate was collected by centrifugation at 10,000× *g* for 10 min at 4 °C. The supernatant was carefully removed and 2 mL of precooled tetrahydrofuran (THF) was added to the pellet, followed by vortexing until the pellet was almost completely dissolved and unstuck from the bottom of the vial. The samples were centrifuged again at 10,000× *g* for 10 min at 4 °C. The supernatant was removed, and the pellet was washed again with 2 mL of THF. The pellet was then dissolved in 50 µL LYSE buffer (Preomics, Planegg-Martinsried, Germany) at 95 °C for 10 min at 1000 rpm. Finally, the sample was digested and processed as described above [30].

The resulting peptides were analyzed using a nano-UHPLC-MS/MS system with an Ultimate 3000 RSLC coupled to an Orbitrap Fusion Tribrid mass spectrometer (Thermo Scientific Instrument, Waltham, MA, USA). Elution was performed with an EASY spray column (75 μm × 25 cm, 2 μm particle size, Thermo Scientific, Waltham, MA, USA) at a flow rate of 400 nl/min using a non-linear gradient of 2–30% solution B (80% acetonitrile and 20% water, 5% dimethyl sulfoxide, 0.1% formic acid) in 60 min. MS analysis was performed in DIA mode. Orbitrap detection was used for MS1 measurements at a resolving power of 120 K in a range between 375 and 1500 *m*/*z* and with 1,200,000 AGC target. Advanced Peak Determination was enabled for MS1 measurements. FAIMS CV was set to -50 at standard resolution. Precursors were selected for data-independent fragmentation in 40 windows ranging from 380 to 980 *m*/*z* with 2 *m*/*z* overlap. HCD collision energy was set to 30%, and MS2 scans were acquired at a resolution of 30 k, 54 ms max. IT, and 500,000 AGC target.

All DIA raw files were processed with Spectronaut version 17 [31] using a library-free approach (directDIA) under the default settings. Enzymes/Cleavage Rules was set to Trypsin/P, LysC. The library was generated against the Uniprot Human database (release UP000005640_9606 November 2022). Carbamidomethylation was selected as a fixed modification, and methionine oxidation, N-terminal acetylation and Deamidation (NQ) were selected as variable modifications. The FDRs of the PSMs and peptide/protein groups were set to 0.01. For quantification, Precursor Filtering was set to Identified (Qvalue), and MS2 was chosen as quantity MS-level.

### 4.4. AKT-mTOR Phosphorylation Pathway Profiling Array

To evaluate AKT-mTOR phosphorylation pathway profiling, we used the Human Phosphorylation Pathway Profiling Array C55 kit (Raybiotech, Peachtree Corners, GA, USA) following the manufacturer’s instructions. Briefly, the membranes were incubated with blocking buffer at room temperature for 30 min and then incubated with 200 μg of cell lysates overnight at 4 °C. The membranes were washed twice with wash buffer and then incubated with Detection Antibody Cocktail at room temperature for 2 h. After washing the membranes twice, they were incubated with horseradish peroxidase-conjugated secondary antibody at room temperature for 2 h. Chemiluminescence signals were acquired and quantified using ChemiDoc and Quantity One software (version 4.6) (Bio-Rad Laboratories, Hercules, CA, USA). Each experiment was performed in triplicate, and intensity differences between treated and untreated conditions were determined and visualized using the Mann-Whitney test and volcano plot. Intensity differences with a two-sided *p* value < 0.05 were considered statistically significant.

### 4.5. PLK1 Phosphorylation Activity

The kinase activity of PLK1 was assessed using a dot-blot assay. Briefly, the nitrocellulose membrane was assembled in dot-blot apparatus (Bio-Rad, Hercules, CA, USA) and 100 µg of everolimus-treated (500 nM) or untreated podocyte cells, solubilized in 25 mM Tris-HCl pH 7.6, 150 mM NaCl, NP40 (1% *v*/*v*), sodium deoxycholate 1% *w*/*v*, and 0.1% *w*/*v* sodium dodecyl sulfate, was applied directly into different well plates. The membrane was blocked with Superblock (ThermoFisher Scientific, Waltham, MA, USA) overnight at 4 °C and washed three times in PBS-T. The samples were then incubated with anti-human pCDC25C S198, or anti-human pTPT1 S46, (Cell Signaling Technology, Danvers, MS, USA) diluted 1:1000 in PBS-T containing 1% *w*/*v* BSA in different wells overnight at 4 °C. The membrane was washed again three times with PBS-T and incubated with HRP-conjugated goat anti-rabbit IgG antibody (Novus Biologicals/Bio-Techne, Minneapolis, MN, USA) diluted 1:5000 in PBS-T containing 1% *w*/*v* BSA for 2 h at room temperature. Chemiluminescence signals were acquired and quantified using ChemiDoc and Quantity One software (version 4.6) (Bio-Rad Laboratories, Hercules, CA, USA). Each experiment was performed in three biological replicates, and differences in intensity between treated and untreated conditions were determined using the Mann-Whitney test. Differences in intensity with a two-sided *p* value < 0.05 were considered statistically significant. HRP-conjugated secondary antibodies and cell lysates not incubated with primary antibodies were used as positive and negative controls, respectively.

### 4.6. Patients

For the validation part of this study, we enrolled 28 kidney transplant recipients receiving everolimus-based immunosuppressive therapy (in combination with mycophenolate mofetil 500 mg b.i.d. and methylprednisolone 4 mg/day) and 8 healthy volunteers matched for the main demographic features. Clinical data are summarized in Table 1. The study was conducted according to the Declaration of Helsinki and was approved by the local Ethics Committee (1745CESC).

### 4.7. Enzyme-Linked Immunosorbent Assay (ELISA)

Human serine/threonine protein kinase (also known as Polo-like kinase 1, PLK1) levels were measured using a homemade direct ELISA in urine samples from the validation cohort. Briefly, Nunc MaxiSorp™ ELISA plates (Thermo Fisher Scientific, Waltham, MA, USA) were coated with 50 µg of urinary samples diluted in carbonate buffer and incubated overnight at 4 °C. Uncoated wells were used for background subtraction. The wells were washed three times with phosphate-buffered saline at pH 7.4 (PBS) and blocked with 3% BSA in PBS overnight at 4 °C. The wells were washed again three times in PBS plus 0.05% *v*/*v* Tween-20 (PBS-T), followed by incubation with 100 µL of mouse monoclonal anti-human PLK1 diluted 1:1000 in PBS-T containing 1% *w*/*v* BSA overnight at 4 °C. The wells were washed again three times with PBS-T and incubated with goat anti-mouse IgG HRP-conjugated antibody diluted 1:2000 in PBS-T containing 1% *w*/*v* BSA for 30 min at room temperature. Finally, the wells were washed three times with PBS-T.

Human osteopontin (SPP1) content was measured in urinary samples from the validation cohort using a commercially available ELISA kit by Proteintech (Planegg-Martinsried, Germany) according to the manufacturer’s instructions. Both assays were developed using TMB substrate (Bio-Rad Laboratories, Hercules, CA, USA), and the reaction was stopped by adding sulfuric acid. The absorbances of both assays were read at 450 nm using the iMark plate reader apparatus (Bio-Rad Laboratories, Hercules, CA, USA).

### 4.8. Statistical Analysis of the Mass Spectrometry Data

After log2 conversion, the identified proteins were filtered for 70% presence in at least one fraction. Normal distribution was used for imputing missing values, and the entire dataset was normalized using the quantile method. The normalized dataset was analyzed with unsupervised hierarchical clustering (multidimensional scaling with k-means) and Spearman’s correlation to identify outliers and dissimilarities between samples. To identify proteins that maximize the discrimination between untreated and treated cells or their supernatants over the everolimus dose, we applied an ANOVA test for paired samples. Then, to identify the proteins that maximized discrimination between the untreated and each everolimus dose, we applied the *t*-test, machine learning methods, such as non-linear support vector machine (SVM), and partial least squares discriminant analysis (PLS-DA). For the ANOVA and *t*-test, proteins were considered statistically significantly expressed for power values of 80% and adjusted *p*-values ≤ 0.05 after correction for multiple interactions (Benjamini-Hochberg method). Volcano plots were used to visualize the *t*-test analysis, establishing the cutoff line using the function y = |c/(x − x0)|. In addition, both the support vector machine (SVM) rank and variable importance in projection (VIP) score were used to identify a priority list of the importance of statistically significant features in each discrimination. In the SVM ranking, the lowest value corresponds to the maximum discriminating power. In contrast, for the VIP score, the highest value corresponds to the maximum discriminating power. Furthermore, in SVM learning, a kernel linear algorithm with a fourfold cross-validation approach was applied to estimate prediction and classification accuracy. The entire matrix was randomly divided into two parts: one for learning (65%) and the other for testing (35%). This partition was repeated until all samples were used for both learning and testing, ensuring a comprehensive evaluation of the model’s performance across the entire dataset. The iterative nature of this process allows us to assess the generalization capabilities of the machine learning model and identify the prediction accuracy. Finally, the confusion matrix was used to identify the minimum number of variables (proteins) needed to distinguish the different clusters studied and to define the most promising ranked list of potential biomarkers for discrimination.

Gene set enrichment analysis (GSEA) was performed to construct a functional protein network based on gene ontology (GO) annotation terms extracted from the Gene Ontology Consortium (http://www.geneontology.org/, released August 2023). The list of all statistically significant proteins from each comparison was loaded into the Joint Pathway Analysis function of the MetaboAnalyR package (version 4.3.3) [32] to identify the protein-associated biochemical pathways that maximize discrimination between each condition. Each pathway with an impact score greater than the corresponding expected value and a two-tailed *p* value < 0.05 after correction for multiple interactions (False Discovery Rate method) was considered statistically significant. Kinase enrichment analysis (KEA) was also performed for statistically significant proteins to identify kinases potentially involved in regulating the phosphorylation signal in each fraction [33]. The same statistically significant cutoff as in the GSEA analysis was used in the KEA analysis. Each kinome tree was visualized using the Coral app [34].

For ELISA, the concordance between everolimus dose and urinary titers of PLK1 or SPP1 was determined using the nonlinear regression fitting equation detector of OriginLab Pro version 2022b software. A correlation coefficient > 0.9 between the experimental data and the predicted equation model was considered significant. Furthermore, a two-tailed *p* value < 0.05 was considered statistically significant. All statistical tests were performed using OriginLab Pro version 2022b and R version 4.3.3 (https://www.R-project.org/) software.

## 5. Conclusions

In conclusion, this study, although mainly performed in an in vitro model for a limited period of treatment, has revealed some potential new mechanisms mediating the side effects of mTOR-Is that could be employed in the future as biological markers to personalize immunosuppressive treatment to minimize kidney allograft toxicity.

## Figures and Tables

**Table 1:** Clinical and demographic characteristics of the kidney transplant recipients receiving maintenance treatment with everolimus (EVE)-based immunosuppressive therapy included in the study.

Characteristics	Values
Number of patients	28
Age (years)	56.04 ± 13.40
Gender (M/F)	16/12
Time since transplantation (years)	11.5 ± 5.68
Serum creatinine (mmol/L)	1.77 ± 0.82
eGFR (mL/min)	55.17 ± 20.91
Daily proteinuria (g/24 h)	0.06 ± 0.11
Trough level EVE (ng/mL)	2.89 ± 1.48

eGFR: Glomerular filtration rate estimated with Nankivell formula. Values are expressed as mean ± Standard Deviation.

## Data Availability

The mass spectrometry data have been deposited in the ProteomeXchange Consortium via the PRIDE partner repository. The computer codes used in the MetaboAnalyst 6.0 package are available at the following link https://github.com/xia-lab/MetaboAnalystR.

## Supplementary Materials

The supporting information can be downloaded at: https://www.mdpi.com/article/10.3390/ijms25137336/s1.

## Abbreviations

mTOR-Is	mTOR inhibitors
mTORC1	mammalian target of rapamycin complex 1
mTORC2	mammalian target of rapamycin complex 2
CNI	calcineurin inhibitors
VEGF	vascular endothelial growth factor
PLS-DA	partial least squares discriminant analysis
SVM	support vector machine
SD	slit diaphragm
VIP	variable importance in projection
GO	gene ontology
KEA	kinase enrichment analysis
EVE	everolimus
PLK1	Polo-Like Kinase 1
SPP1	osteopontin
NPHS1	nephrin
NPHS2	podocin
SYNPO	synaptopodin
PBS	phosphate-buffered saline
BSA	bovine serum albumin
EVs	extracellular vesicles
