# Supplementary material for: Proteomic Changes Induced by the Immunosuppressant Everolimus in Human Podocytes
Source: Int J Mol Sci. 2024 Jul 4;25(13):7336. doi: 10.3390/ijms25137336 (PMC11242170; doi:10.3390/ijms25137336)
Supplement: Supplementary file 1 [file ijms-25-07336-s001.zip › Bruschi et al_Supplementary Figures.pdf]

Supplementary Figures

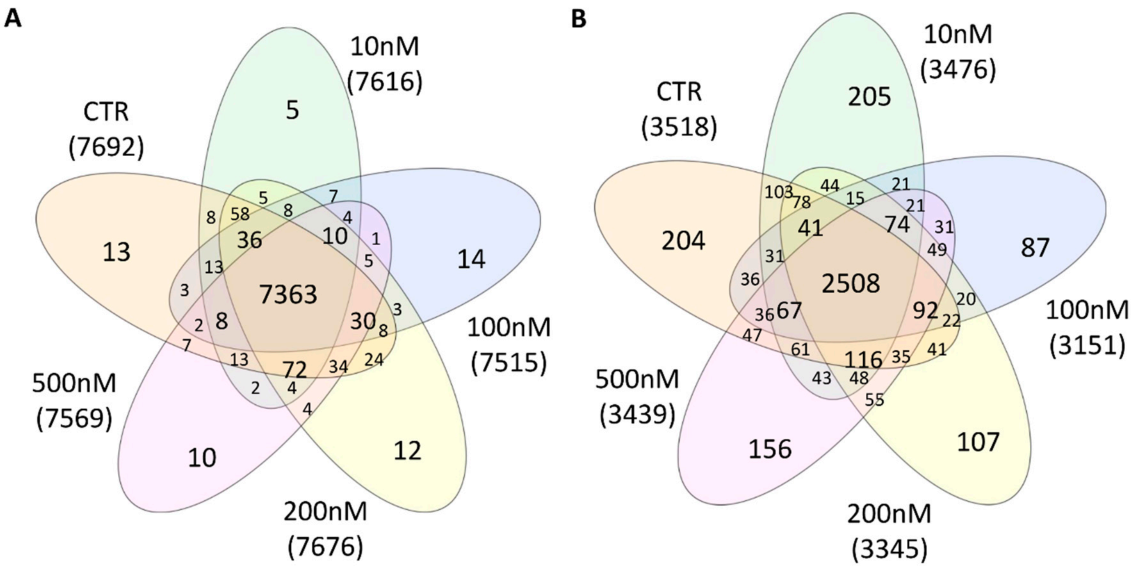

Figure S1. Venn diagram of the total proteins identified in (A) podocytes and (B) supernatants. Venn diagram showing the common and exclusive proteins identified. Numbers and circles represent distinct proteins under each condition.

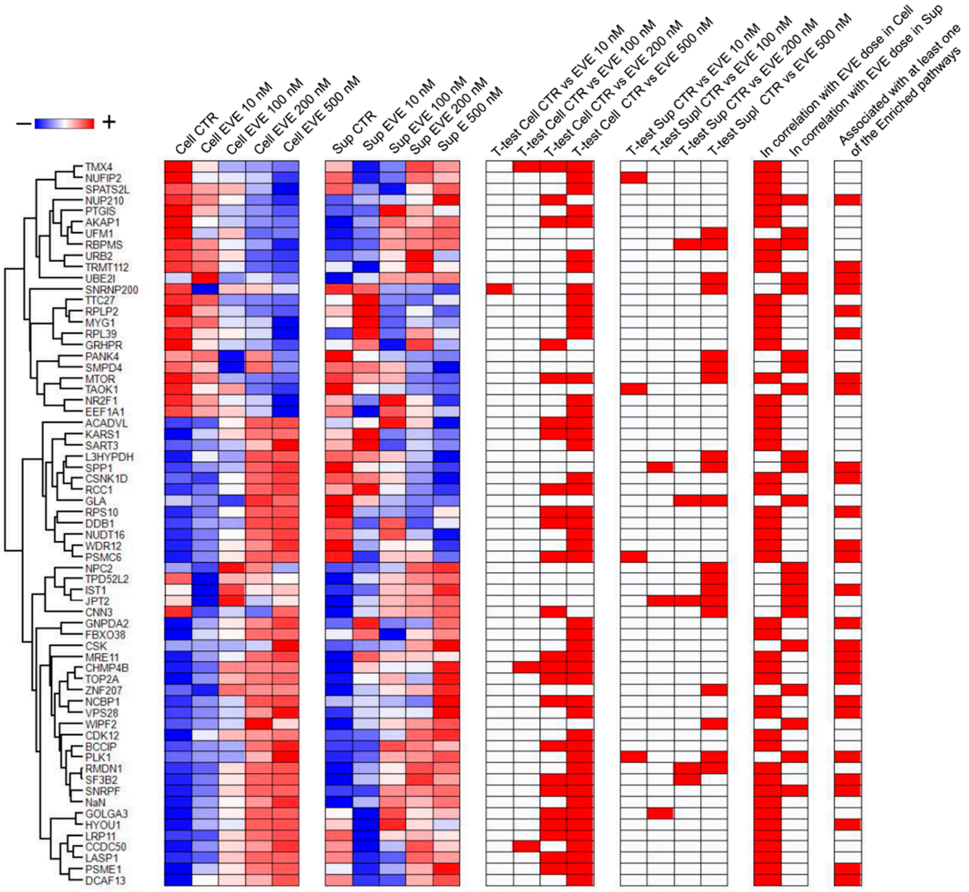

Figure S2. Heatmap of 65 statistically significant proteins correlated with everolimus dose. In the heatmap, each row represents a protein and each column corresponds to a group condition. Normalized Z-scores of protein abundance are depicted by a pseudocolor scale with red indicating positive expression, white equal expression, and blue negative expression compared to each protein value. The dendrogram displays the outcome of unsupervised hierarchical clustering analysis, placing similar protein profile values near each other (see detail in Supplementary Table S1).

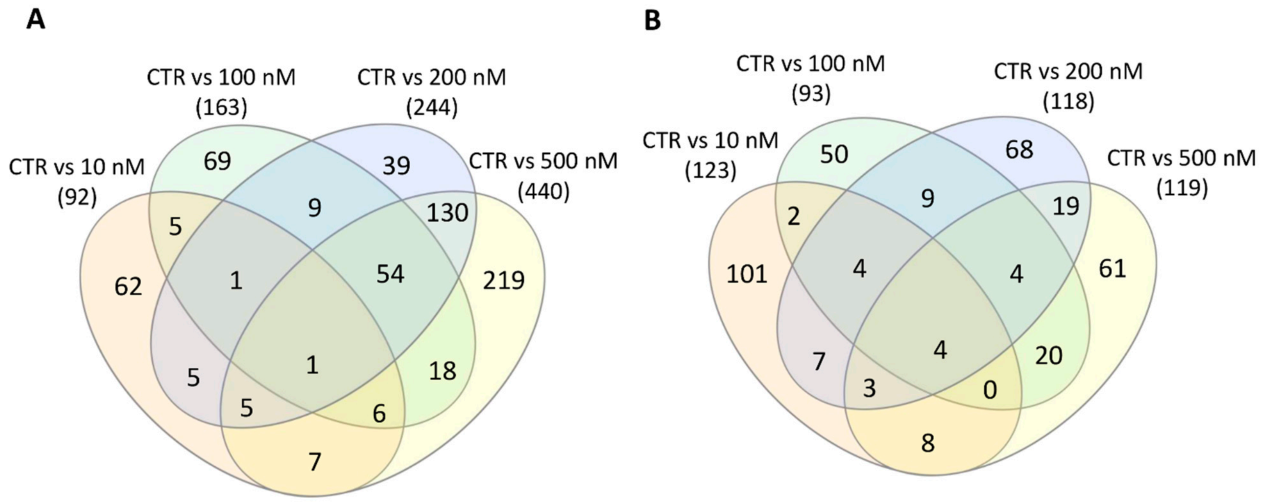

Figure S3. Venn diagram of all statistically significant proteins identified in (A) podocytes and (B) their supernatants using t-test. Venn diagram showing the common and exclusive proteins identified. Numbers and circles represent distinct proteins under each condition.

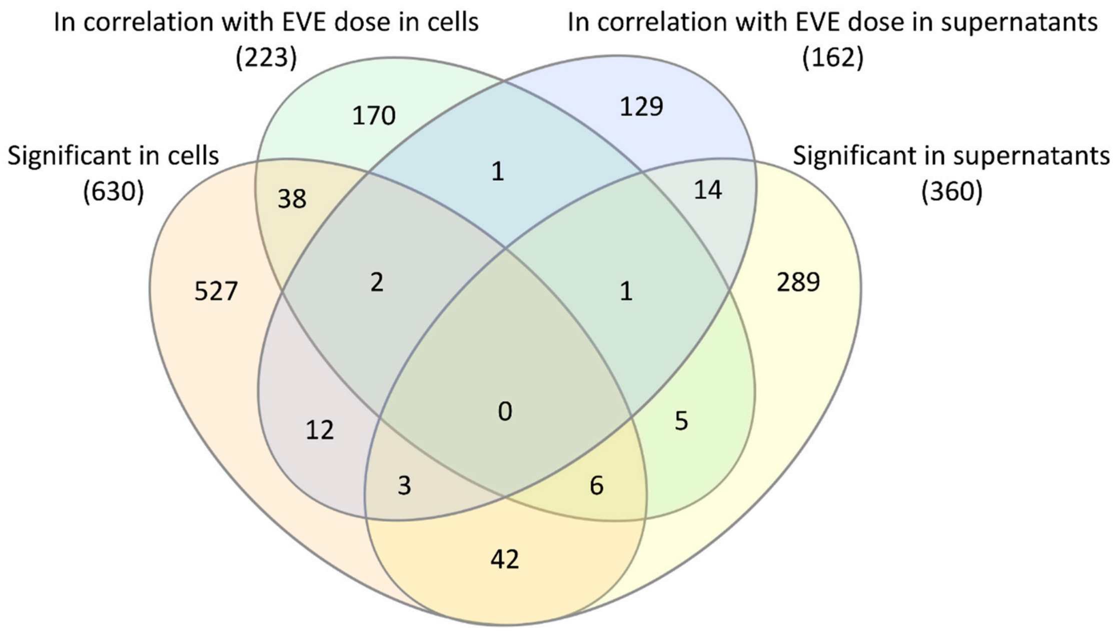

Figure S4. Venn diagram of all statistically significant proteins correlated with everolimus dose. Venn diagram showing common and exclusive proteins identified using ANOVA in cells and their supernatant samples. Numbers and circles represent distinct proteins under each condition.

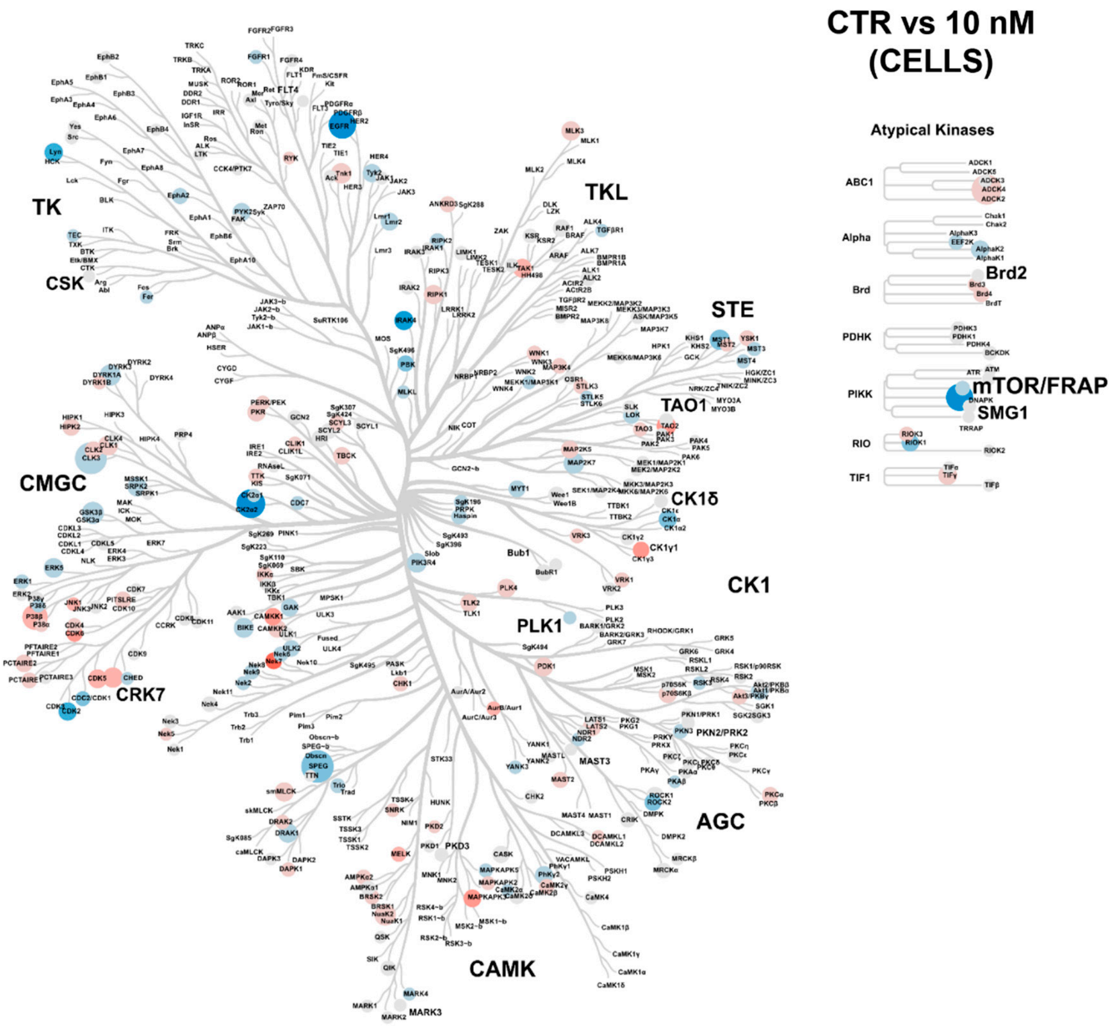

Figure S5. Kinase tree diagram generated by the Coral app for comparing cells untreated and treated with 10 nM everolimus. Each circle represents an identified kinase. Log_2 kinase fold change is depicted by a pseudocolor scale with red indicating overexpression, white equal expression, and blue underexpression in treated samples. The circle size is proportional to the corresponding $-Log_{10}$ P-value in the t-test analysis.

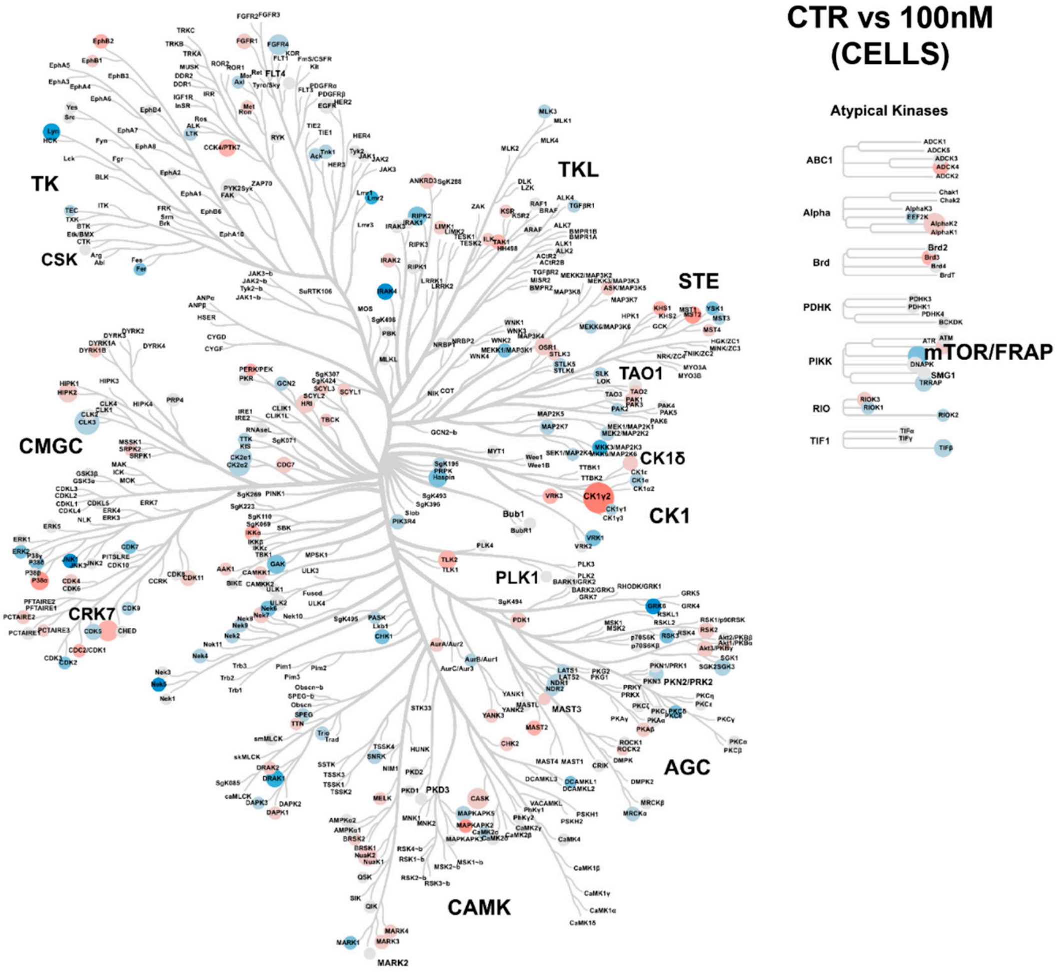

Figure S6. Kinase tree diagram generated by the Coral app for comparing cells untreated and treated with 100 nM everolimus. Each circle represents an identified kinase. Log_2 kinase fold change is depicted by a pseudocolor scale with red indicating overexpression, white equal expression, and blue underexpression in treated samples. Circle size is proportional to the corresponding $-Log_{10}$ P-value in t-test analysis.

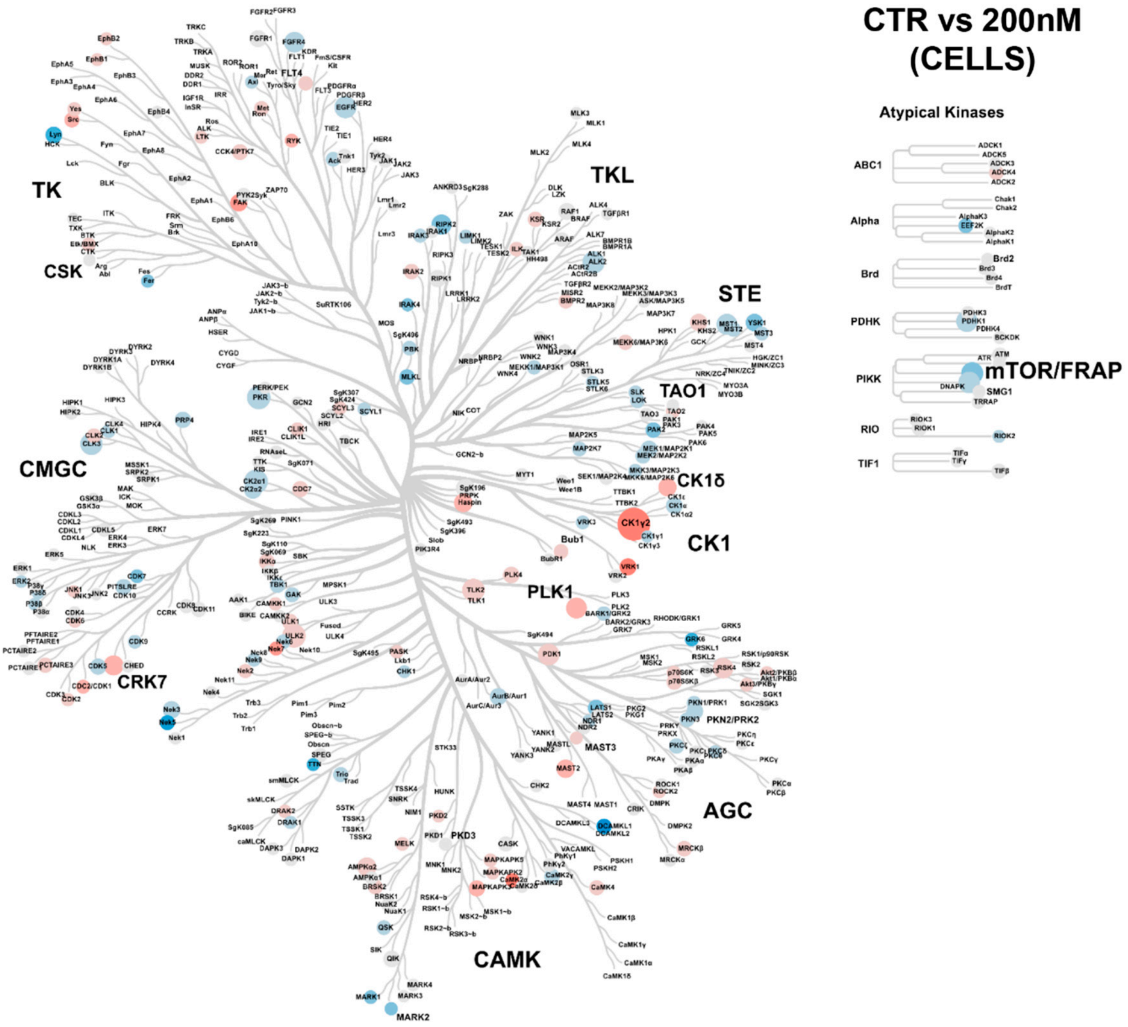

Figure S7. Kinase tree diagram generated by the Coral app for comparing cells untreated and treated with 200 mM everolimus. Each circle represents an identified kinase. Log_2 kinase fold change is depicted by a pseudocolor scale with red indicating overexpression, white equal expression, and blue underexpression in treated samples. The circle size is proportional to the corresponding $-Log_{10}$ P-value in the t-test analysis.

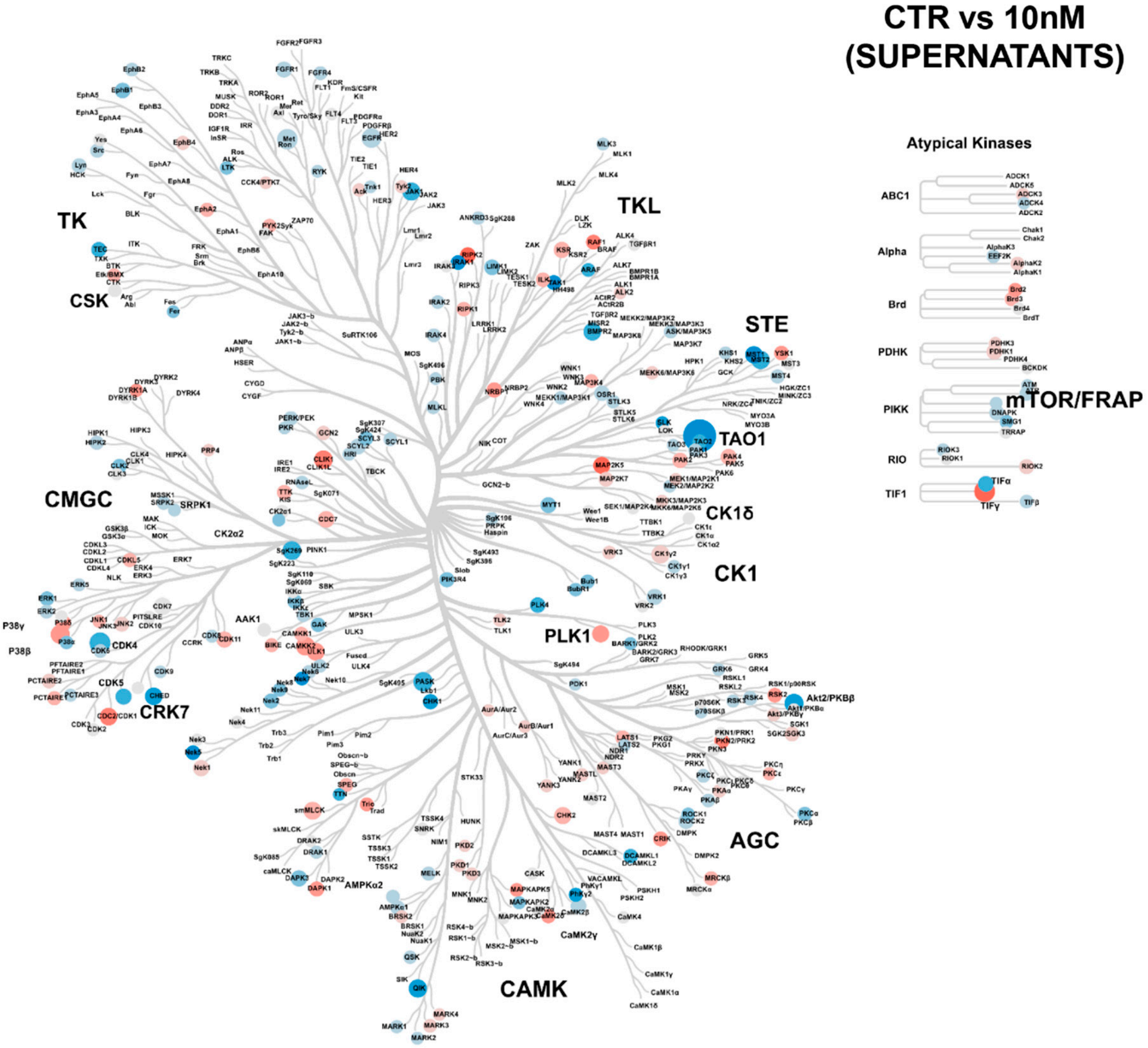

Figure S8. Kinase tree diagram generated by the Coral app for comparing the supernatants of cells untreated and treated with 10 nM everolimus. Each circle represents an identified kinase. Log_2 kinase fold change is depicted by a pseudocolor scale with red indicating overexpression, white equal expression, and blue underexpression in treated samples. The circle size is proportional to the corresponding $-Log_{10}$ P-value in the t-test analysis.

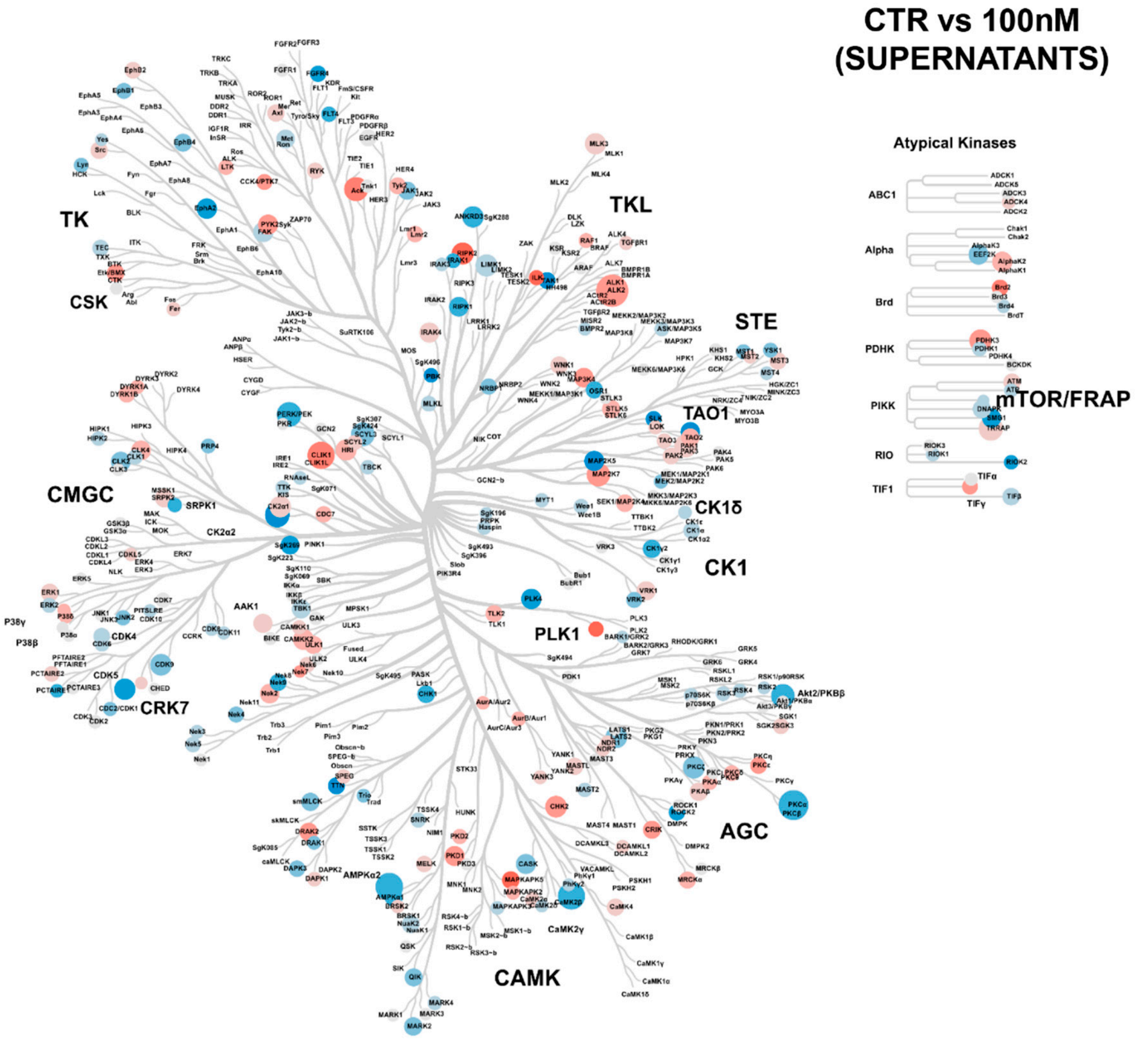

Figure S9. Kinase tree diagram generated by the Coral app for comparing the supernatants of cells untreated and treated with 100 nM everolimus. Each circle represents an identified kinase. Log_2 kinase fold change is depicted by a pseudocolor scale with red indicating overexpression, white equal expression, and blue underexpression in treated samples. The circle size is proportional to the corresponding $-Log_{10}$ P-value in the t-test analysis.

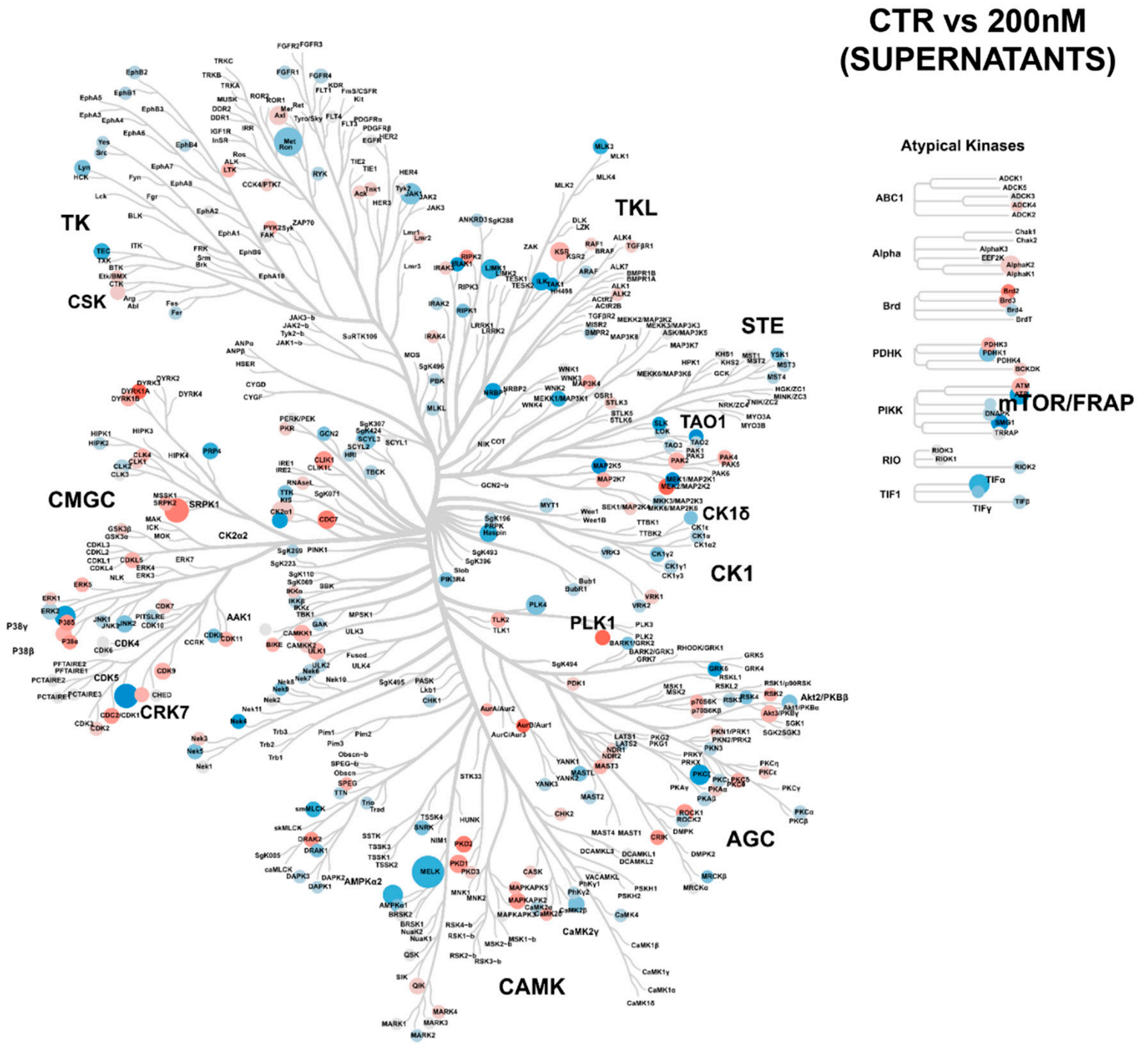

Figure S10. Kinase tree diagram generated by the Coral app for comparing the supernatants of cells untreated and treated with 200 nM everolimus. Each circle represents an identified kinase. Log_2 kinase fold change is depicted by a pseudocolor scale with red indicating overexpression, white equal expression, and blue underexpression in treated samples. The circle size is proportional to the corresponding $-Log_{10}$ P-value in the t-test analysis.

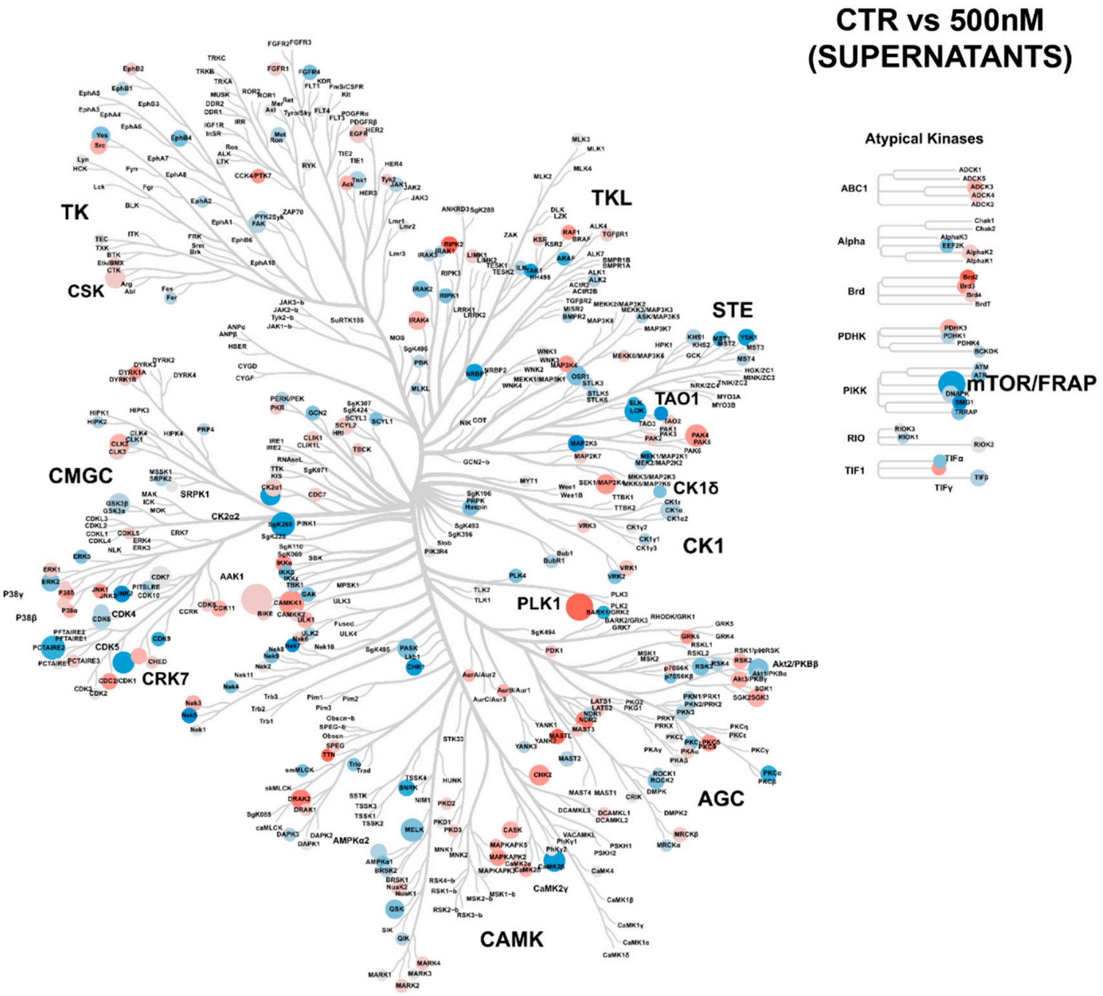

Figure S11. Kinase tree diagram generated by the Coral app for comparing the supernatants of cells untreated and treated with 500 nM everolimus. Each circle represents an identified kinase. Log_2 kinase fold change is depicted by a pseudocolor scale with red indicating overexpression, white equal expression, and blue underexpression in treated samples. The circle size is proportional to the corresponding $-Log_{10}$ P-value in the t-test analysis.

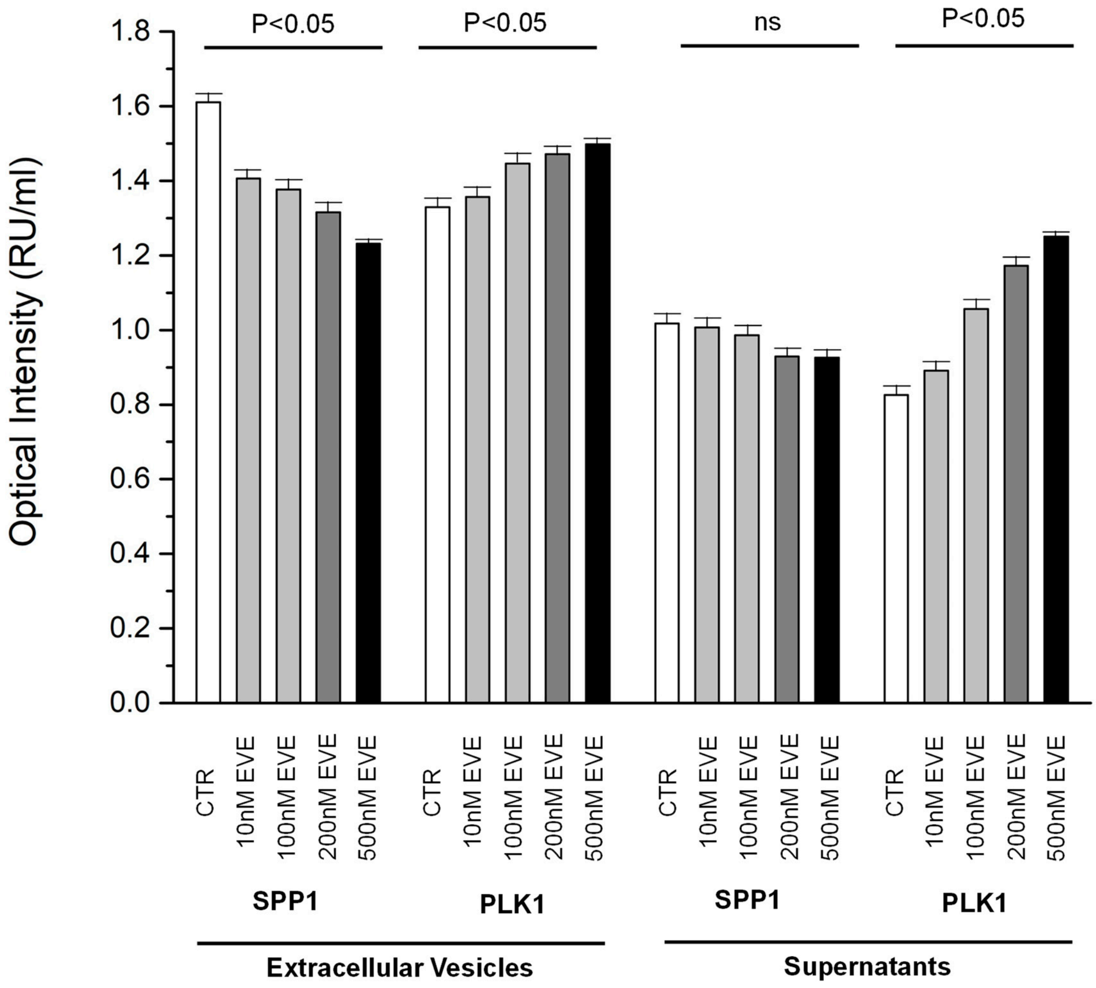

Figure S12. PLK1 and SPP1 contents in extracellular vesicles and supernatants of untreated and everolimus (EVE)-treated podocytes. The content of PLK1 and SPP1 was measured by ELISA. PLK1 was significantly increased (P<0.05) in both supernatants and extracellular vesicles of EVE-treated cells compared to the CTR. Contrarily the content of SPP1 was reduced by the EVE treatment in a dose-dependent manner. P value by ANOVA.

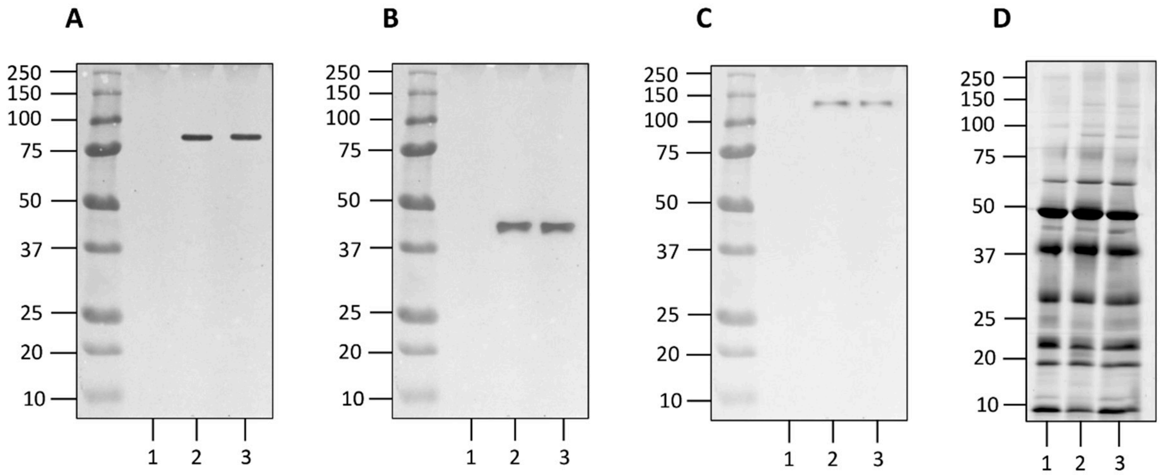

Figure S13. Representative western blot analysis of full-length gel (8-16T%) for **(A)** Synaptopodin, **(B)** Podocin, and **(C)** Nephrin proteins in the whole lysate of untreated human podocytes (lines 2, 3). The 3 antibodies detected a single band corresponding to the predicted molecular weight of each protein. The fibroblast cell line was used as negative control (line 1). **(D)** Silver– blue staining of the same samples was used as loading control.

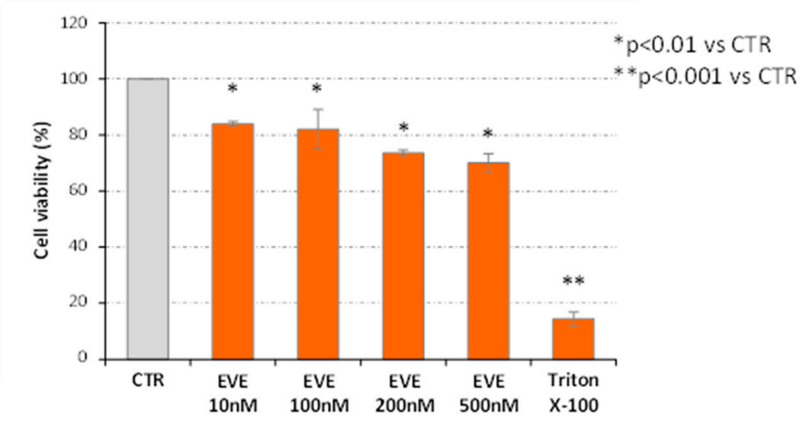

Figure S14. The cytotoxic effect of everolimus (EVE) on podocytes. Cells were treated with increasing concentrations of everolimus for 24 h. The mTOR-I inhibited cell growth in a dose dependent manner. Triton X-100 was used as positive control. *$p<0.01$; **$p< 0.001$ vs CTR by T-test.

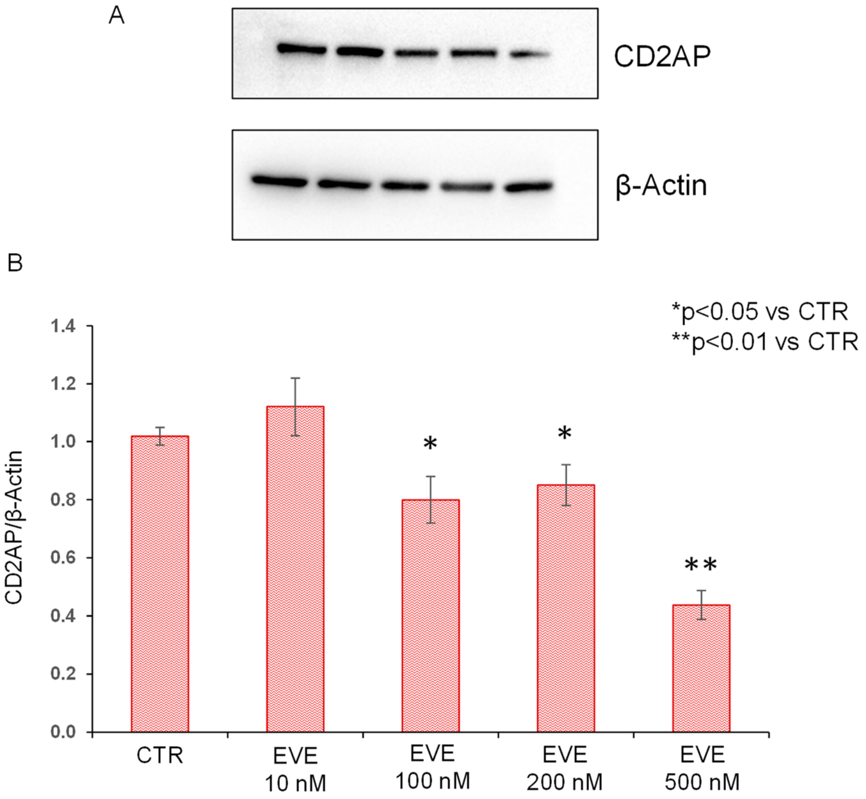

Figure S15. CD2AP protein expression in podocytes treated with EVE. (A) Representative images of protein blots. **(B)** The expression level of CD2AP. The bars show the mean expression in arbitrary units (±SD). *$p < 0.05$, **$p < 0.001$ vs CTR, Student's t-test.
