# Supplementary material for: Proteomic Changes Induced by the Immunosuppressant Everolimus in Human Podocytes
Source: Int J Mol Sci. 2024 Jul 4;25(13):7336. doi: 10.3390/ijms25137336 (PMC11242170; doi:10.3390/ijms25137336)
Supplement: Supplementary file 1 [file ijms-25-07336-s001.zip › Bruschi et al_Supplementary Methods.pdf]

Supplementary Methods

MTT assay

The inhibitory effect of everolimus on the viability of podocytes was measured by the MTT assay. Cells (1×10^3) were seeded in each well of a 96-well plastic culture plate and left overnight in complete medium. The cells were then treated with the intended doses of everolimus for 24 h. After the incubation period, MTT was added to a final concentration of 0.5 mg/ml and the cells were incubated for 4 h. Dimethyl sulfoxide was added for reaction and the absorbency was measured at 595 nm with microplate reader FilterMax F5 (Molecular Devices, San Jose, CA, USA). The experiments were performed three times independently, in triplicate each time and the average values of the three independent experiments were calculated.

Western blot

Expression of main podocyte cells markers were evaluated by western blot. 30 μg of whole lysate of untreated podocyte cells were solubilized in 2% w/v SDS, 10% glycerol and 62.5 mM Tris-HCl pH 6.8, separated by sodium dodecyl sulfate polyacrylamide gel electrophoresis (SDS-PAGE), and then transferred to a nitrocellulose membrane. After blocking with 3% (w/v) bovine serum albumin (BSA) in PBS containing 0.05% v/v Tween-20 (T-PBS) the membrane was incubated with the following primary antibodies: anti-nephrin (Santa Cruz Biotechnology, Dallas TX, USA, 1:1000, sc-19000), anti-podocin (Santa Cruz Biotechnology, Dallas TX, USA, 1:1000, sc-21009), or anti-synaptopodin (Santa Cruz Biotechnology, Dallas TX, USA, 1:1000, sc-21537). After rinsing in T-PBS, the membrane was incubated with HRP-conjugated secondary antibodies (diluted 1:10000 in 1% w/v BSA in PBS-T). Chemiluminescence signal was acquired and quantified using respectively the ChemiDoc and Quantity One software (Bio-Rad, Hercules, CA, USA). Fibroblast cells was used as negative control and blue silver staining of same samples was used as loading control. Gel electrophoresis was digitized by GS-800 Densitometer (Bio-Rad, Hercules, CA, USA).

Extracellular vesicles (EVs) isolation

Extracellular vesicles (EVs) were isolated from the supernatants of untreated and EVE-treated podocytes according to the protocol in our previous study [bruschi,] with some modifications. Briefly, after collecting supernatants, the samples were centrifuged at 3,000xg for 15 minutes at 4°C to remove cells and debris. Then, the supernatants were centrifuged at 100,000xg for 120 minutes at 16°C. To obtain a clean EVs fraction, the pellet was washed five times in PBS and centrifuged at 100,000 × g for 10 min at 4°C. The samples were stored at -80°C until use.

ELISA for PLK1 and SPP1 in extracellular vesicles (EVs) and supernatants

The secretion of PLK1 and SPP1 proteins was evaluated by ELISA. Briefly, aliquots of 5 μg of cell supernatants (Sup) and their EVs obtained from untreated and treated podocytes were solubilized in 25 mM Tris-HCl pH 7.6, 150 mM NaCl, 1% v/v NP40, 1% w/v sodium deoxycholate, 0.1% w/v sodium dodecyl sulfate, and added to Nunc MaxiSorp™ ELISA well (Thermo Fisher Scientific, Waltham, MA, USA). After overnight incubation at 4°C samples were removed and wells were blocked with 3% BSA in PBS overnight at 4°C. Wells were washed three times in PBS-T followed by incubation with 100 μl of mouse monoclonal anti-human PLK1 (Biorbyt, Cambridge, United Kingdom) or mouse monoclonal anti-human SPP1 (Santa Cruz Biotechnology, Dallas, TX USA) diluted 1:1000 in PBS-T containing 1% w/v BSA overnight at 4°C. Wells were washed again three times with PBS-T and incubated with goat anti-mouse IgG HRP-conjugated antibody (Novus Biologicals/Bio-Techne, Minneapolis, MN, USA) diluted 1:2000 in PBS-T containing 1% w/v BSA for 30 min at room temperature. Finally, the wells were washed three times with PBS-T. The assays were developed with TMB substrate (Bio-Rad Laboratories, Hercules, CA, USA), and then stopped by adding sulfuric acid. The absorbance of both tests was read at 450 nm using an iMark plate reader (Bio-Rad Laboratories, Hercules, CA, USA). The results were expressed as Relative Unit for ml (RU/ml). Each sample was run in triplicate. Histogram plot was used to visualize the optical intensity.
