# Supplementary material for: Proteomic Changes Induced by the Immunosuppressant Everolimus in Human Podocytes
Source: Int J Mol Sci. 2024 Jul 4;25(13):7336. doi: 10.3390/ijms25137336 (PMC11242170; doi:10.3390/ijms25137336)
Supplement: Supplementary file 1 [file ijms-25-07336-s001.zip › Bruschi et al_Supplementary Results.pdf]

Supplementary Results

Evaluation of the release mechanism of PLK1 and SPP1 proteins

To evaluate the release mechanism of PLK1 and SPP1 proteins from untreated and everolimus-treated podocytes a direct ELISA was used. Figure S2 showed that PLK1 content was significantly increased ($P<0.05$) in both supernatants and extracellular vesicles of EVE-treated cells compared to the CTR. Contrarily the content of SPP1 was reduced by the EVE treatment in a dose-dependent manner (Figure S2).
